# Interfractional target changes in brain metastases during 13-fraction stereotactic radiotherapy

**DOI:** 10.1186/s13014-021-01869-4

**Published:** 2021-07-28

**Authors:** Megumi Uto, Kengo Ogura, Tomohiro Katagiri, Keiichi Takehana, Takashi Mizowaki

**Affiliations:** 1 Department of Radiation Oncology and Image-Applied Therapy, Kyoto University Graduate School of Medicine, 54 Shogoin Kawahara-cho, Sakyo-ku, Kyoto, 606-8507 Japan; 2 Department of Radiation Oncology, Japanese Red Cross Wakayama Medical Center, Wakayama, Japan; 3 Department of Radiation Oncology, Shizuoka City Shizuoka Hospital, Shizuoka, Japan

**Keywords:** Brain metastases, Fractionated stereotactic radiotherapy, Interfractional target changes, Adaptive radiotherapy

## Abstract

**Background:**

The risk for radiation necrosis is lower in fractionated stereotactic radiotherapy (SRT) than in conventional radiotherapy, and 13-fraction SRT is our method of choice for the treatment of brain metastases ≥ around 2 cm or patients who are expected to have a good prognosis. As 13-fraction SRT lasts for at least 17 days, adaptive radiotherapy based on contrast-enhanced mid-treatment magnetic resonance imaging (MRI) is often necessary for patients undergoing 13-fraction SRT. In this study, we retrospectively analyzed interfractional target changes in patients with brain metastases treated with 13-fraction SRT.

**Methods:**

Our analyses included data from 23 patients and 27 metastatic brain lesions treated with 13-fraction SRT with dynamic conformal arc therapy. The peripheral dose prescribed to the planning target volume (PTV) was 39–44.2 Gy in 13-fractions. The gross tumor volume (GTV) of the initial SRT plan (initial GTV), initial PTV, and modified GTV based on the mid-treatment MRI scan (mid-treatment GTV) were assessed.

**Results:**

The median initial GTV was 3.8 cm^3^ and the median time from SRT initiation to the mid-treatment MRI scan was 6 days. Compared to the initial GTV, the mid-treatment GTV increased by more than 20% in five lesions and decreased by more than 20% in five lesions. Interfractional GTV volume changes of more than 20% were not significantly associated with primary disease or the presence of cystic components/necrosis. The mid-treatment GTV did not overlap perfectly with the initial PTV in more than half of the lesions.

**Conclusions:**

Compared to the initial GTV, the mid-treatment GTV changed by more than 20% in almost one-third of lesions treated with 13-fraction SRT. As SRT usually generates a steep dose gradient as well as increasing the maximum dose of PTV compared to conventional radiotherapy, assessment of the volume and locational target changes and adaptive radiotherapy should be considered as the number of fractions increases.

## Background

Metastatic brain tumors are common, occurring in 9.6% of cancer patients, according to the Metropolitan Detroit Cancer Surveillance System [1]. The treatment of brain metastases is similar to that of extracranial cancers, consisting of a combination of surgery, systemic therapy, and radiotherapy.

The development of novel systemic therapies has dramatically improved the prognosis of patients with brain metastases, and neurocognitive dysfunction and late toxicities due to whole brain radiotherapy (WBRT) are significant concerns for long-term survivors [2]. The implementation of stereotactic radiosurgery (SRS) instead of WBRT reduces the risk for neurocognitive dysfunction and brain atrophy, and SRS is currently the method of choice for patients with a limited number of brain metastases (i.e., 1–3 metastatic lesions) [3, 4]. In addition, JLGK0901 study showed that overall survival did not differ between patients with five to ten brain metastases and those with two to four tumors [5]. Therefore, SRS for up to ten brain metastases has become one of the treatment options.

Fractionated stereotactic radiotherapy (SRT) has emerged as a promising approach to treat brain metastases, with a lower risk of brain necrosis [6–8]. Although the optimal fractionation number remain unclear in SRT for brain metastases, it seems that the toxicities of SRT are reduced, as the number of fractionation increases. Therefore, at our institution, to reduce the risk of brain necrosis while providing similar local control, we typically use a 13-fraction SRT in patients with brain metastases who have a good prognosis or lesions ≥ around 2 cm.

SRT generates steep dose gradient. Kubo et al. reported the MRI appearance change during SRT [9], and target volume change during SRT should be considered to achieve local control and decrease the risks of toxicities. As 13-fraction SRT lasts at least 17 days, we conduct adaptive re-planning as necessary, based on contrast-enhanced mid-treatment magnetic resonance imaging (MRI). However, interfractional target changes in brain lesions during 13-fraction SRT remain unclear. In addition, it is also unknown whether the histology of primary disease or the presence of cystic components/necrosis are related to the interfractional target change. Furthermore, the information of the interfractional target change could contribute to consider the needs of conducting adaptive re-planning. Moreover, that information seems useful in not only 13-fraciton but also other fractionated SRT regimens. Therefore, we think that it is important to assess the interfractional target change and analyze the clinical characteristics, locational relationship, and dose-volume histogram. In this study, we retrospectively analyzed the clinical data of patients with brain metastases undergoing 13-fraction SRT to determine the interfractional target changes in brain metastases.

## Methods

### Patient population

Between July 2015 and November 2018, 37 patients with brain metastases were treated with 13-fraction SRT at our institution. Patients treated with postoperative radiotherapy to the surgical cavity or single isocenter volumetric-modulated arc therapy for multiple brain metastases, as well as those without available contrast-enhanced mid-treatment MRI scans, were excluded. Our analyses included data from 23 patients with 27 brain metastases treated with 13-fraction SRT with dynamic conformal arc therapy. The characteristics of the study cohort are shown in Table 1. This study was conducted in accordance with the 1964 Declaration of Helsinki, and all procedures were approved by the institutional ethical review board (R1048). Written informed consent was obtained from all patients.

**Table 1:** Patient characteristics

Age	Median, 70 years (range 42–85 years)
Sex (male/female)	19/4
Karnofsky Performance Status	Median, 90 (range 50–100)
Primary tumor (number of brain metastases)	
Lung cancer	15 (19)
Renal cell carcinoma	3 (3)
Others	5 (5)
Prior whole-brain radiotherapy	2 cases (25 Gy/10 fr., 30 Gy/10 fr.)
Cystic component or necrosis (yes/no)	19/8
Treatment device per lesion	
TrueBeamSTx/Vero4DRT	25/2
Prescribed dose	
Prescribed to isocenter	4 lesions with 48.75 Gy/13 fr. (PTV was almost covered by 80% isodose line of the prescribed dose)
D_99.5%_ = 100%	23 lesions with 39–42 Gy/13 fr. (Dmax: almost 125% of the prescribed dose)

*PTV* planning target volume, *Dmax* maximum dose

### Contouring and treatment planning for brain metastases

Patients were immobilized using thermoplastic masks, and computed tomography (CT) images (thickness of 1–1.25 mm) were acquired using a Light Speed RT scanner (GE Healthcare, Milwaukee, WI, USA). Contouring and treatment planning were performed using iPlan RT version 4.5 (BrainLab AG, Munich, Germany). Contrast-enhanced MRI scans were fused with the planning CT images using a calculation grid size of 1 mm and the calculation algorithm Monte-Carlo. The planning target volume (PTV) was determined by adding a 1 mm margin to the gross tumor volume (GTV), defined as the volume of the contrast-enhancing lesion. The peripheral dose prescribed to the PTV was 39–44.2 Gy delivered in 13 fractions. Twenty-five lesions were treated with TrueBeamSTx (Varian Medical Systems, Palo Alto, CA, USA), and two lesions were irradiated using Vero4DRT (Hitachi, Ltd., Tokyo, Japan).

We delivered 3–3.4 Gy per fraction to the periphery of the PTV in 13 fractions because more than 8 fractions are required to maximally get the benefit of fractional irradiation such as reoxygenation phenomenon [10]. In addition, PTV is created by adding a 1 mm margin to the GTV considering the interfractional changes, and the peripheral doses to the GTV are estimated at around 42.9–48.6 Gy. Moreover, the center doses of GTV are 48.8–55.3 Gy.

As for the size of brain metastases in 13-fraction SRT, lesions ≥ around 2 cm were treated by 13-fraction SRT. It was because that the rate of local recurrence and radionecrosis in brain metastases > 2.0 cm was higher compared to lesions ≤ 2 cm in single-fraction SRS [11]. In addition, the incidence of radiation necrosis in lesions with a maximum diameter of ≤ 2.0 cm was 17%, and the incidence of radionecrosis was larger as time passed from SRS [12, 13]. Thus, we also applied 13-fraction SRT in patients who were expected to have good prognosis even if the size of lesions was smaller than 2 cm in diameter.

### Changes in GTV and relationship with initial PTV

**Fig. 1:**
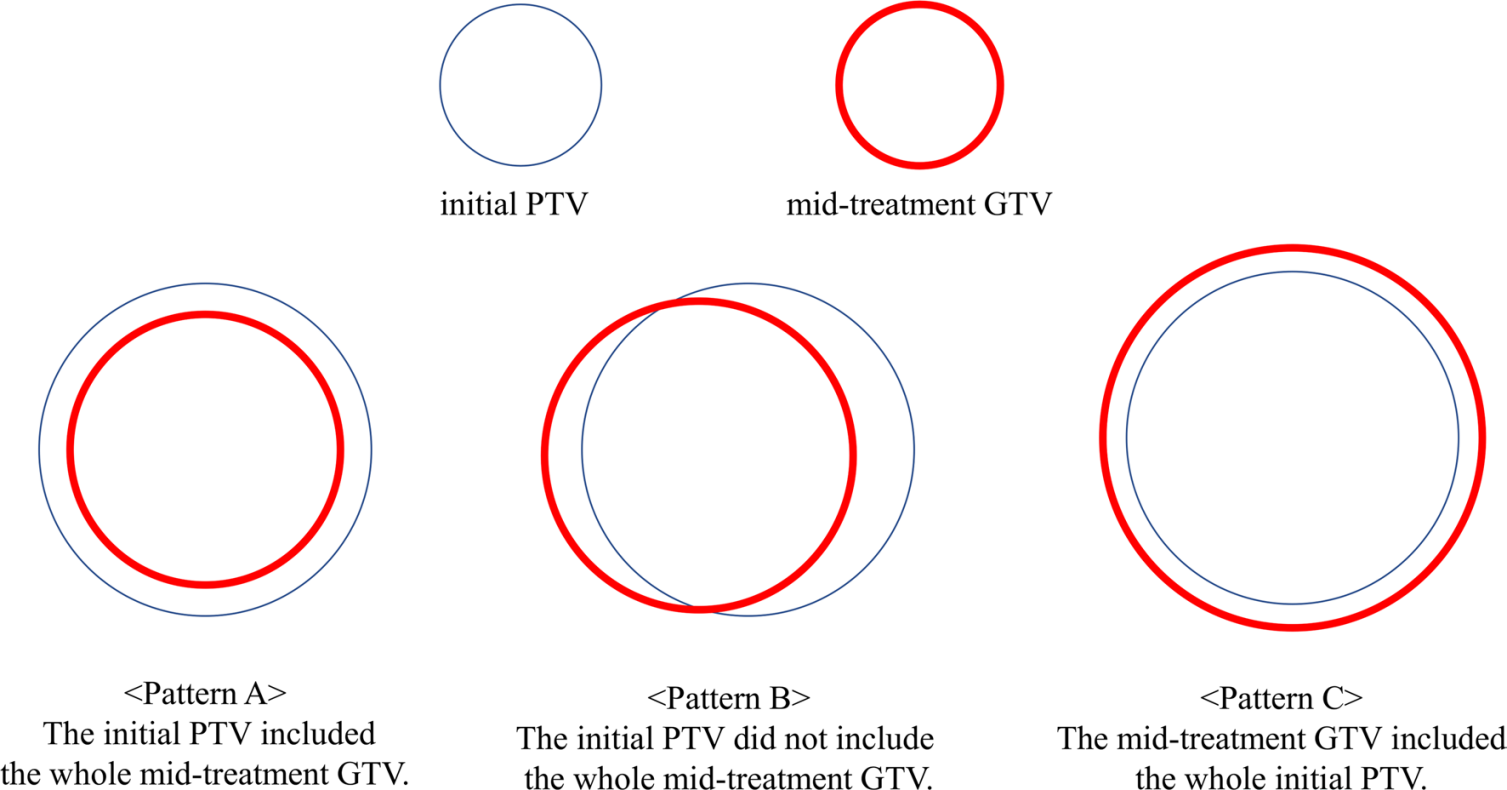
Three patterns of the relationship between the initial PTV and the mid-treatment GTV

We calculated and compared the GTV of the initial SRT plan (initial GTV) with the modified GTV based on the mid-treatment MRI scan (mid-treatment GTV). The mid-treatment MRI scan was merged with the planning CT images. To assess the need for adaptive re-planning, we investigated the overlap between initial PTV and the mid-treatment GTV. We classified the locational relationship of initial PTV and mid-treatment GTV into three patterns (Fig. 1): pattern A, when the initial PTV included the whole mid-treatment GTV; pattern B, when the initial PTV did not include the whole mid-treatment GTV; and pattern C, when the mid-treatment GTV included the whole initial PTV. There are no strict criteria for conducting adaptive re-planning. When considering re-planning, we evaluate not only any target volume change but also target migration and the dose distribution evident on mid-treatment MRI.

We also analyzed the D_2%_, D_50%_, and D_98%_ of the mid-treatment GTV in the initial SRT plan, where D_2%_, D_50%_, and D_98%_ were the doses covering 2%, 98%, and 50% of the mid-treatment GTV, respectively. Because the radiation dose was prescribed to the initial PTV, irradiated doses to the initial GTV differed for each lesion. Therefore, we calculated the differences in the irradiated dose between the initial GTV and mid-treatment GTV (Diff_midGTV_DXX%_) as follows: Diff_midGTV_DXX%_ = {GTV_DXX%_(mid-treatment) − GTV_DXX%_(initial)} × 100. GTV_DXX%_(mid-treatment) was the irradiated dose covering XX% of the mid-treatment GTV in the initial SRT plan, and GTV_DXX%_(initial) was the irradiated dose covering XX% of the initial GTV. Diff_midGTV_D2%_, Diff_midGTV_D98%_, and Diff_midGTV_D50%_ were evaluated in this study.

### Statistical analyses

All statistical analyses were performed using EZR, a graphical user interface for R (the R Foundation for Statistical Computing, Vienna, Austria, version 3.4.1) [14]. EZR is a modified version of R commander version 2.4–0, facilitating biostatistical evaluations. The relationships among interfractional GTV changes, the presence of cystic component or necrosis, and local recurrence were analyzed using the Fisher’s Exact test. *P* values < 0.05 were considered statistically significant.

## Results

### Interfractional GTV changes

**Fig. 2:**
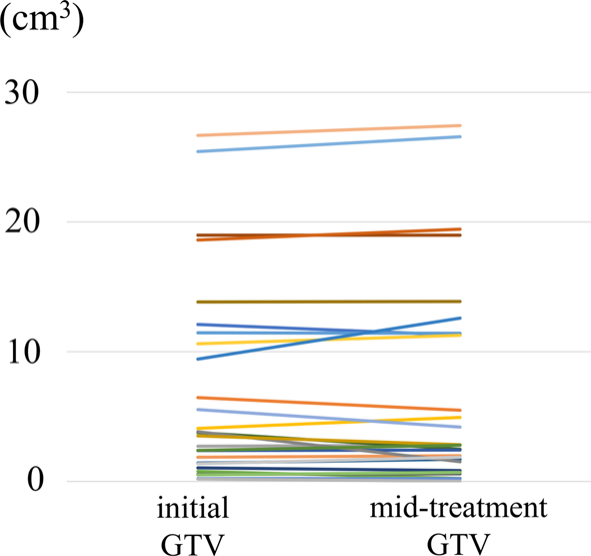
The initial GTV and the mid-treatment GTV

**Fig. 3:**
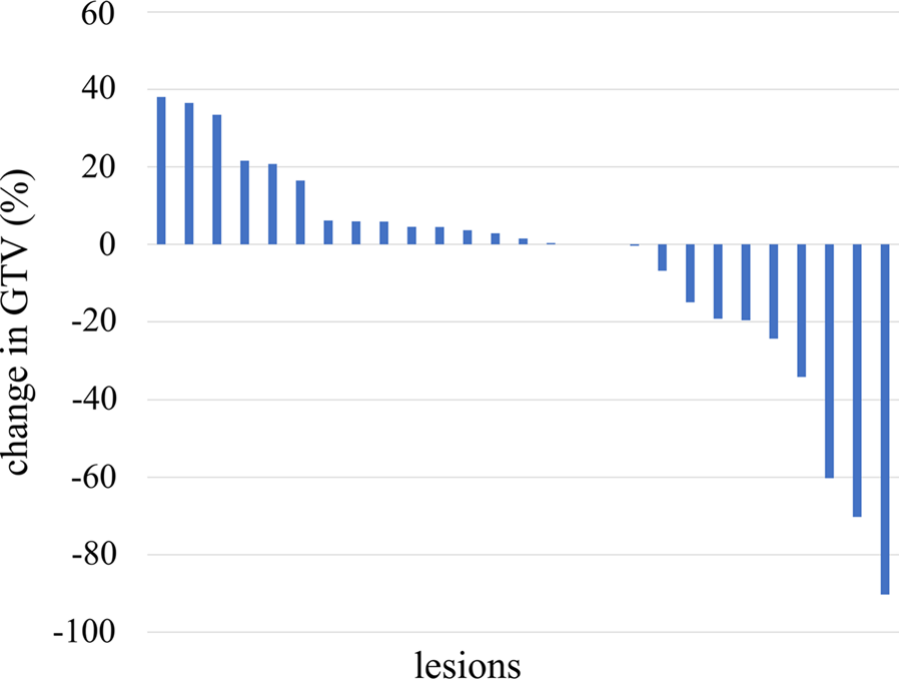
Waterfall plot showing changes in GTV

The median initial GTV was 3.8 cm^3^ (range 0.2–26.7 cm^3^). As shown in Table 2, the median time from the start of SRT to the mid-treatment MRI scan was 6 days (range 3–11 days). Relative to the initial GTV, the mid-treatment GTV increased by more than 20% in five metastatic lesions and decreased by more than 20% in five metastatic lesions. There was no significant association between interfractional GTV changes of more than 20%, primary disease (lung cancer vs. other cancers), or the presence of cystic components/necrosis (Table 3). The interfractional changes between the initial GTV and mid-treatment GTV are shown in Figs. 2 and 3.

**Table 2:** The time from SRT initiation or CTS to the mid-treatment MRI scan

Definition	Median period (days)
From SRT initiation to the mid-treatment MRI scan	6 (range 3–11)
From CTS to the mid-treatment MRI scan	8 (range 6–17)
From the MRI scan before CTS to the mid-treatment MRI scan	14 (range 7–28)
From the MRI scan before CTS to SRT initiation	8 (range 3–21)

*SRT* stereotactic radiotherapy, *MRI* magnetic resonance imaging, *CTS* CT simulation

**Table 3:** Associations between interfractional change, primary tumor, and adaptive re-planning

	Lung cancer	Others	*P* value (Fisher’s test)
GTV change < 20%	12	5	0.363
GTV change > 20%	9	1	
No adaptive re-planning	8	5	0.077
Adaptive re-planning	13	1	

	No re-planning	Re-planning	*P* value
GTV < 20%	11	6	0.046
GTV change > 20%	2	8	

	Solid	Cystic component or necrosis	*P* value
GTV < 20%	5	12	1
GTV change > 20%	3	7	

*GTV* gross tumor volume

### Association between the initial PTV and the mid-treatment GTV

Out of 27 metastatic lesions, the initial PTV included the mid-treatment GTV in 12 lesions (pattern A). In the remaining 15 lesions, the initial PTV did not include the whole mid-treatment GTV (pattern B). None of the lesions exhibited a pattern C association between the initial PTV and the mid-treatment GTV.

### Irradiated dose to the mid-treatment GTV in the initial SRT plan

**Fig. 4:**
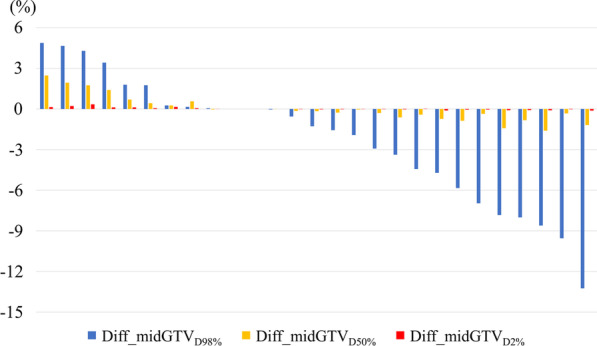
Waterfall plot of dose differences between the initial GTV and mid-treatment GTV. Diff_midGTV_DXX%_ was defined as {GTV_DXX%_(mid-treatment) minus GTV_DXX%_(initial)} × 100. GTV_DXX%_(mid-treatment) was the radiation dose covering XX% of the mid-treatment GTV in the initial SRT plan, and GTV_DXX%_(initial) was the radiation dose covering XX% of the initial GTV

The Diff_midGTV_D2%_, Diff_midGTV_D50%_, and Diff_midGTV_D98%_ are shown in Fig. 4. The columns in each target were sorted in descending order of Diff_midGTV_D98%_. The changes in Diff_midGTV_D98%_ were larger compared to the changes in Diff_midGTV_D2%_ and Diff_midGTV_D50%_. Diff_midGTV_D2%_ was small in all lesions.

## Discussion

We analyzed the interfractional GTV changes during 13-fraction SRT using mid-treatment MRI scans. Interfractional GTV changes of over 20% were observed in 37% of the brain lesions. We found no significant associations between interfractional GTV changes of more than 20%, primary disease, or the presence of cystic components/necrosis. In more than half of the lesions, the initial PTV did not include the whole mid-treatment GTV. To the best of our knowledge, this is the first study to report interfractional target changes during 13-fraction SRT for brain metastases. In addition, this is the first report to analyze the relationship between the interfractional target volume change and the histology of primary disease or the presence of cystic component/necrosis. Moreover, the mid-treatment GTV partly protruded from the initial PTV in more than half of the lesions. Therefore, clinicians should consider adaptive re-planning in patients with brain metastases undergoing 13-fraction SRT.

SRT is often the treatment of choice for patients with brain metastases because the risk for late toxicities and brain necrosis is relatively low. As the number of fractions increases to reduce the risk of radiation necrosis, the dose per fraction must decrease too. We use a 13-fraction SRT in patients with brain metastases to reduce the risk of brain necrosis while providing similar local control. It is because that the reoxygenation utilization rate was higher as the number of fractions increased [10] and Minniti et al. showed better local control and a reduced risk of radionecrosis in multi-fraction SRS compared to single-fraction SRS [7]. In addition, Jiang et al. reported high local control for brain metastases larger than three centimeters by using SRT with median prescribed dose as 40 Gy in median 10 fractions [6]. Numerous studies have confirmed that SRT offers satisfactory local control with a lower risk of radiation-induced brain necrosis [7, 8, 15]. In patients with brain metastases, the number of fractions in SRT is typically less than 11, and the total treatment period is short; thus, reports on brain metastases volume changes during treatment are limited [9, 16–18]. Hessen et al. [16] analyzed the differences between MR0 (MRI before SRT) and MR1 (MRI during SRT) in patients with brain metastases undergoing SRT with the number of fractions ranging from three to five. They found that the median tumor volume in MR0 was 9.5 cm^3^, whereas the tumor volume in MR1 was 12.2 cm^3^. They also reported dose coverage impairments due to the changes in the target volume [16]. Similarly, Salkeld et al. [17] reported changes in the target volume during radiosurgical planning. They also showed that the median PTV increased from 4.14 to 4.47 cm^3^ during treatment planning [17]. The target volume may change during the preparation and planning of 13-fraction SRT because the median time from the MRI scan before CT simulation (CTS) to the SRT initiation was 8 days. The risk of target volume change should be considered. Therefore, we contoured the target volume on contrast-enhanced CT images for CTS, if not medically contraindicated, to minimize the risk of target volume change before SRT commencement. Consistently, Kubo et al. [9] reported target volume changes during 5–8 fraction SRT for large brain metastases. Collectively, the findings of these studies suggest that the target volume may increase during SRT and that changes in the target volume may be affected by the time required for SRT planning and quality assurance. In this study, we evaluated interfractional target volume changes during 13-fraction SRT and found that the volume of five lesions decreased by more than 20% compared to the pre-treatment MRI volume. The antitumor effects of 13-fraction SRT might have contributed to the target shrinkage.

In contrast to conventional radiotherapy, SRT typically entails steep dose gradients and delivery of high maximum doses to the PTV. Thus, adaptive radiotherapy (i.e., re-planning) should be considered as the number of fractions in SRT increases. The mid-treatment GTV partly protruded from the initial PTV in more than half of the lesions; hence, brain lesions should be monitored for locational deviations and volume changes during SRT. In addition, it is difficult to detect the locational change by reference to only a target volume change. We classified the target migration into three patterns (A, B, and C of Fig. 1). Even if the mid-treatment GTV shrinks dramatically during 13-fraction SRT, migration may be triggered by a change of surrounding edema, and the migration type may change, for example, from the A to B. It is important to check not only target volume change but also the dose distribution evident on the mid-treatment MRI when considering adaptive re-planning.

Moreover, we found that Diff_midGTVD_98%_ was less than 0% in more than half of the lesions. This finding implies that insufficient doses may be delivered to the mid-treatment GTV based on the initial SRT plan, possibly leading to a high risk of local recurrence. Of note, Diff_midGTVD_98%_ was more than 3% in four lesions. High Diff_midGTVD_98%_ may increase the radiation dose delivered to the surrounding normal tissue; thus, patients should be closely monitored for toxicities when the Diff_midGTVD_98%_ is high.

There were some limitations to our study. First, the study was retrospective, and the number of patients was relatively small. Second, we did not assess the relationship between changes in the target location and edema in the surrounding tissues. As changes in the location of the target may arise from cerebral edema, it is critical to evaluate whether the initial prescribed dose is sufficient to cover the entire lesion [18]. Finally, we did not take into consideration the history or timing of systemic therapy in our analyses. Similar to radiotherapy, systemic therapy is likely to affect the interfractional target volume.

## Conclusions

We analyzed the interfractional GTV changes during 13-fraction SRT using mid-treatment MRI scans and found that the mid-treatment GTV changed by more than 20% in almost one-third of the metastatic brain lesions. In more than half of the lesions, the mid-treatment GTV did not overlap perfectly with the initial PTV. To the best of our knowledge, this is the first study to assess interfractional target changes during 13-fraction SRT. As SRT involves steep dose gradients and delivers higher doses to the PTV than conventional radiotherapy, adaptive radiotherapy should be considered, particularly in patients receiving a high number of fractions.

## Data Availability

The data that support the findings of this study are available on request from the corresponding author.

## Abbreviations

CT: Computed tomography
CTS: CT simulation
Diff_midGTV_DXX%_: {GTV_DXX%_(mid-treatment) − GTV_DXX%_(initial)} × 100
GTV_DXX%_(initial): Irradiated dose covering XX% of the initial gross tumor volume
GTV_DXX%_(mid-treatment): Irradiated dose covering XX% of the mid-treatment gross tumor volume in the initial stereotactic radiotherapy plan
initial GTV: Gross tumor volume of the initial stereotactic radiotherapy plan
initial PTV: Planning target volume of the initial stereotactic radiotherapy plan
GKS: Gamma Knife radiosurgery
GTV: Gross tumor volume
mid-treatment GTV: Modified gross tumor volume based on the mid-treatment magnetic resonance imaging scan
MRI: Magnetic resonance imaging
PTV: Planning target volume
SRS: Stereotactic radiosurgery
SRT: Stereotactic radiotherapy
WBRT: Whole brain radiotherapy
